# Type 2 Diabetes Prevalence, Distribution and Risk Factors in St. Kitts and Nevis, West Indies

**DOI:** 10.23937/2377-3634/1410114

**Published:** 2019-11-28

**Authors:** Jeanita W Richardson, Kelsie D Kelly, Trina K Kumodzi, Nicole Slack Liburd, Hazel Laws

**Affiliations:** 1 Department of Public Health Sciences, University of Virginia, USA; 2 School of Nursing, University of Virginia, USA; 3 Country Programme Specialist, Pan American Health Organisation (PAHO), USA; 4 Chief Medical Officer, Federation of St. Kitts & Nevis, Basseterre, St. Kitts, West Indies

## Abstract

**Objectives:**

This study in collaboration with the St. Kitts & Nevis Ministry of Health (MOH) investigated the prevalence of type 2 diabetes and its risk factors, the second leading cause of death in the country.

**Methods:**

Medical records of patients between the ages of 18 and 75 as of January 1, 2010 treated between January 1, 2010 and December 31, 2015 in the government-funded health centers (HCs) were eligible for inclusion (N = 2737). All HC visits (n = 4169) generated by a representative sample of patients (n = 761) were evaluated using Caribbean Public Health Association Public Health Association (CARPHA) guidelines for risk ranges.

**Results:**

The prevalence of type 2 diabetes is 23% and is concentrated in the 45–64 year-old cohort. Though women outnumber men 3:1 in the sample, gender-prevalence rates are similar (23% and 22% respectively). There is also evidence that comorbidities are prevalent among diabetics (76%) and many nondiabetics are at risk of diabetes (20%).

**Conclusion:**

This study confirmed the MOH’s concerns that type 2 diabetes presents local risk and brings into question historic assumptions that women are at greater risk of diabetes than men.

## Introduction

International and regional health organizations concur that the direct and indirect costs of diabetes mellitus (hereafter referred to as diabetes or type 2 diabetes) are unsustainable [1–4]. While the prevalence of type 2 diabetes is a worldwide phenomenon, its burdens are particularly acute for persons of African and Caribbean descent and citizens of low- and middle income countries [5–8]. The focus on type 2 diabetes prevention, diagnosis and management is supported by two factors, prevalence and preventability. Type 2 diabetes accounts for 90% of cases of global diabetic cases and 95% of cases in the Caribbean [1–3].

Important tools in identifying risk are determining whether a first-degree relative has the disease (parent or sibling); regular metabolic and cardiovascular screenings, which identify impaired glucose tolerance and risk such as, cholesterol and triglyceride levels; as well as, hypertension and excessive weight [1–3,9].

Costs associated with supporting this growing chronically ill population include but are not limited to, increased use of health services, lost work productivity, disabilities and fiscal burdens of monitoring and managing glucose levels among patients, their families, communities and national economies. Medical expenditures for patients with diabetes are 2–3 times higher than persons without the chronic disease [2]. Some re- searchers have estimated the direct (health care) and indirect (lost productivity, premature mortality and quality of life) costs of diabetes in the English Caribbean alone exceed $1 billion USD [4]. Currently, with three of four diabetics living in “low - to middle-income countries” it is clear that the lifelong health system burdens associated with the disease portends significant resource drains on economies [2].

The International Diabetes Federation (IDF), the Pan American Health Organization (PAHO/a division of the World Health organization), CARICOM (a formal Carib- bean state collaborative with 20 member countries), as well as the Caribbean Public Health Agency (CARPHA) have all identified the Caribbean as a region of concern for non-communicable diseases (NCDs) in general and diabetes in particular [10,11]. As per a 2016 CARICOM report, NCDs account for 40% of deaths in populations younger than 70 years of age. Hypertension is the leading cause of death followed by diabetes and cardiovascular disease. The diabetes prevalence in the region is double the global rates (8.5%). It has also been reported that women at least in part because of the prevalence of overweight are at higher risk of acquiring diabetes [12,13].

Supporting these concerns are findings of Caribbean-focused diabetes research. The risk of acquiring the disease is more prevalent among Afro-Caribbeans than any other African-ethnic group. The likelihood of a diabetes diagnosis increases with age, most notably after 65-years-old. Of the research available focusing on the English-speaking Caribbean, Jamaica and Trinidad and Tobago are the most frequent sites [14].

If there is good news about diabetes it is that the perpetuation of current trends are not inevitable. Trends can be halted and even reversed with a data-driven public health prevention focus [1,15]. Unfortunately, for the decision makers of the Federation of St. Kitts and Nevis, prior to this study there was virtually no published research nor local systematic assessment of the state of diabetes specific to the Federation.

The two-island Federation of St. Kitts and Nevis has a population in excess of 52,000 with approximately 11,000 residing in Nevis [1,13]. Demographics of the Federation have changed in recent years with 27% of the population under the age of 15 and 10% of the population over 60 [16]. A federal system is articulated in the Constitution with parallel health ministries on both islands, because Nevis has autonomous responsibilities in domestic matters. The Federation’s MOH allocates significant resources to the universal health care system, which includes free standing clinics (six in Nevis and 11 in St. Kitts) and hospitals (3) with a focus on preventing disease and early detection, as opposed to tertiary prevention which focuses on minimizing disability and effects of the disease [13,15,17].

In an effort to assess the period prevalence of type 2 diabetes and confirm or dispel perceptions of the burden of diabetes, the Ministry of Health, in partnership with the University of Virginia developed a research plan to create baseline data to inform diabetes preventative and management interventions.

## Methods

This retrospective study, using patient charts sought to identify the distribution and period prevalence rate of diabetes in St. Kitts and Nevis, was approved by the University of Virginia IRB Board, the St. Kitts and Nevis Ministry of Health and the Federation’s Interim Research Review Board (IERC). Confidentiality agreements were signed by all involved in data collection and analysis. Study inclusion criteria were persons between the ages of 18–75 as of January 1, 2010 who had also visited the clinic at least once between January 1, 2010 and December 31^st^ 2015.

Once the complete list of eligible files was compiled and alphabetized at each health center the selection was as follows; every other chart was selected for inclusion in clinics with 200 or fewer eligible participants; every third record in clinics with 201–400 eligible participants; and, every fifth record in clinics with 401 or more eligible participants. Relevant names were assigned unique ID numbers.

In research teams of two, relevant files were pulled and the assigned ID number was placed on the top of the paper sheet where every visit’s information between 2010–2015 was recorded. Paper chart review sheets included gender, health markers and metabolic screenings known to be risk factors for diabetes, such as cholesterol levels, fasting blood glucose, HBA1c, blood pressure, hypertension diagnosis, family history of diabetes. Categorization of risk was defined by CARPHA standards (Table 1). Not all of these measurements are assessed consistently in adult clinical interactions in St. Kitts and Nevis. Furthermore, LDL, and HDL were recorded at much lower rates than combined cholesterol (CC). As a result, CC was used in the analysis.

Readings across patient files covered varied dates across five years indicating period prevalence rates. If multiple readings were available for the same health marker, the mode was used for purposes of analysis. A first degree relative with diabetes was defined as a parent or sibling with a formal diagnosis.

Excel was used to evaluate the data because it is a software accessible to the Ministry and allows for additional local analysis of raw data. Relative to confidentiality, while in St. Kitts and Nevis, the ID patient sheets were kept in a locked safe separate from the locked safe that housed the chart review sheets. Prior to leaving St. Kitts and Nevis all spreadsheets and ID number assignment sheets that contained patient names were destroyed.

Some of the significance of the research effort can be asserted from the proportion of the population represented in the study. The Nevis population as per the last census was approximately 11,000 and 44,000 in St. Kitts. The eligible population of participants (given the inclusion criteria) represented 5% of Federation’s population. The chart review sample represented 1% of the nation’s population. The Federation’s adult population is approximately 36,000 making the sample 2% of all adults [2,11,13,18,19].

## Results

The study assessed 761 patient records from nine St. Kitts and Nevis health centers (HCs). All six of the health centers in Nevis and three of the 11 St. Kitts centers were included in the study. Every discrete health center visit was recorded (n = 4,169) between the years of 2010–2015. The Nevis HC charts did not always record the same data as St. Kitts HCs. For comparison purposes, results discussed reflect the data consistently reported on both islands. Given the limitations of personnel and time the three health centers identified as study priorities by the MOH in St. Kitts serve rural, urban and suburban populations. It should also be noted that six St. Kitts participant charts were not gender specific. None of the 6 patient charts with unclear gender identification were diagnosed with diabetes or hypertension. Their information was not included in gender-specific discussions but were included in overviews of the Federation and each island.

As noted earlier, the Federation of St. Kitts and Nevis is a two-island state. Data revealed nuances between the populations of Nevis and St. Kitts even though the islands are only approximately two miles apart. The largest participant age cohort was 45–64 years-old, representing 37% of the total sample. The second largest cohort differed by island. In the case of Nevis, it was 35–44 year-olds (22%) and the 25–34 (18%) age cohort in St. Kitts. Women are overrepresented as 70% of the Federation sample (70% of Nevis and 67% of St. Kitts charts). Table 2 displays the distribution of ages and genders of the charts reviewed.

The period prevalence of diabetes in the Federation was 23% (n = 173). Persons between 45–64 years of age account for 57% of patients with diabetes even though they represented 37% of the total sample. Secondary age cohorts diagnosed with diabetes varied by island. For example, the 45–64 age group contributed 53% of the diabetic cases followed by the 35–44 age group in Nevis. In St. Kitts the 45–64 age group represented 60% of diabetic cases followed by the 25–34 age group. File notations of first degree relatives with diabetes totaled 148. The remaining 612 files had no notation at all, which could mean the question was not asked or the answer was no. So as not to leave this important risk factor out of the analysis we included the affirmative responses for those with and without diabetes (Table 3 and Table 4).

Table 3 reflects the numbers of diabetic patients with elevated (risk) screenings as set forth by the Caribbean Association of Public Health (CARPHA). The co-morbidity risk screening most consistently reported for diabetics was elevated blood pressure (56%). At the Federation level, 76% of patients with diabetes simultaneously manage a hypertension diagnosis, some of whom struggle to maintain “normal” blood pressure readings as captured below. This diabetes-hypertension comorbidity rate is similar on both islands (77% in Nevisian diabetics and 76% in Kittisian diabetics). Risk factors that can complicate diabetes management in Nevis diabetics were most often elevated blood pressure and FBG readings (89% and 64% respectively). For St. Kitts patients, their heightened risk was reversed, (31% for FBG and 44% for BP).

As noted earlier, risk of acquiring type 2 diabetes increases when a first-degree relative has the chronic disease. Among all diabetics, 25% reported a first-degree relative who had also been diagnosed with diabetes. Again, there was a difference in reporting between Nevis and St. Kitts (41% and 13% of the diabetic population respectively). As reflected in Table 3, not only are persons with diabetes apt to be managing hypertension, but over 50% struggle to keep blood pressure and fasting blood glucose levels in normal ranges. It should be noted that not every person with a type 2 diabetes diagnosis had evidence of an HBA1c test between 2010–2015.

Patient charts did not document all risk screenings for all patients. For example, of the 761 patients in the sample, 191 had FGB’s values, 72 with HbA1c’s, 170 with combined cholesterol. Rather than omit this data, the Ministry of Health wanted as much data as possible analyzed. Relative to comorbidities, 132 (76%) of those with a diabetes diagnosis managed the formally diagnosed comorbidity of hypertension. Table 3 and Table 4 indicate the risk ranges available for the patients in each category with elevated mode readings over the five year period irrespective of any diagnosed comorbidity.

When considering the percentage of diabetic cases by gender it became apparent that males and females seeking health care at HCs have similar rates of diabetic diagnoses. For example, in the national aggregate, the total rate of diabetes in males and females was 22% and 23% respectively. When considering each island separately Nevis gender distribution was 17% of males and females were diabetic. In St. Kitts, 29% of males and 32% of females were diabetic.

Given the structure of the Federation health system, there is also value in assessing HC usage patterns of diabetics. At the national level, visits tied to diabetic patients generated 48% of health center visits. In Nevis, 37% of the 1,680 visits evaluated were generated by diabetic patients (n = 75). In St. Kitts, 55% of 2,489 visits were generated by diabetics (n = 98).

### Undiagnosed diabetes patient risk

Fifteen percent (15%) of persons without diabetes (n = 588) have been diagnosed with hypertension. As was the case with diabetics, the age range most prevalent in this sample was 45–64 (32%). In a repeat of diabetic patient results, the largest Nevisian age cohort was 45–64 (29%) followed by 35–44 year-olds (23%). Kittitians between 45–64 made up 35% of the non-diabetic population followed by 25–34 year-olds (22%). Women outnumber men as non-diabetics [n = 402 (69%) and n = 180 (31%) respectively] which is influenced by their proportion of the sample.

Of those with a hypertension and no diabetes diagnosis (113), only 18 had normal FGB, HbA1c and CC. However, it is important to note that of all of the 113 only hypertensive patients only 46 had FGB values in their charts, 10 had HbA1C and 48 had CC. Given the costs of screenings, which required exporting blood samples to another island, it is possible that comorbidities and elevated risk measure are under reported. Disaggregating by island, 15% of nondiabetic Nevisians and 29% of nondiabetics were closely related to someone with diabetes.

### Limitations

There are a number of limitations that should to be taken into account as the Ministry of Health seeks to address diabetes. Weight or more specifically body mass index (BMI), though imperfect, as per the literature is one of the most cost efficient gauges of diabetes risk. Unfortunately, weight nor BMI were consistently recorded in health records. It is not clear whether the absence of a notation to the question of first degree relatives with diabetes should be interpreted as no or not asked. As a result, we focused on the charts that noted an affirmative response to first degree relatives. Charts are currently handwrit- ten and as such from time to time were difficult to decipher. This challenge was mitigated by input from clinic nurses and multiple members of the research team inspecting the record. The Nevis HC charts did not always record the same data as St. Kitts HCs. For comparison purposes, results discussed reflect the data consistently reported on both islands which precluded assessing more of the research-based risk predictors (e.g. education status, LDL, HDL, nutritional status and physical activity). The males in the sample (30% of 761) do not approximate their proportion of the Federation population (approximately 50%), which may suggest an under-representation of men with or at risk of acquiring diabetes. Laboratory tests such as HBA1c, FBG and cholesterol do not appear to be included in standardized care. Costs associated with these tests may influence the frequency they are ordered. Eight health centers in St. Kitts were not included in the study because of the limitations of time. However, the selection of three health centers representative of the urban, rural and suburban residential areas was informed by the Ministry of Health. As such, it is possible that the health profile in St. Kitts might differ from the results of the three included centers.

### Discussion and Recommendations

Findings of this first-of-its kind evaluation of health records confirm the Ministry of Health’s hypothesis that the numbers of diabetes cases and their age distribution warrant concern. The period prevalence of type 2 diabetes in the St. Kitts and Nevis sample (23%) far exceeds Caribbean predictions of diabetes (9%) and worldwide prevalence (8.3%) as per IDF [5,10].

Findings also reveal the trend of younger than historically expected diabetes diagnoses (65+) [13]. The IDF reports that these trends will remain in low and middle income countries such as St. Kitts and Nevis, where 2 in 3 people with diabetes live in urban areas [10,13]. Furthermore, the IDF reports that the largest age cohort of diabetics are between the ages of 40 and 59 [13]. The age cohort with the highest prevalence of diabetes and who represent the largest proportion of the sample are 45–64 year-olds, followed by different age groups in Nevis and St. Kitts with diabetes cases in all younger age cohorts. This suggests specific population targets for prevention education and screening for each island.

Potential stresses to the Federation’s health system are further taxed because of comorbidities. For example, 76% of diabetics in the sample were also hypertensive. Furthermore, those with diabetes tended to have difficulty managing their non-communicable diseases (NCDs) or NCD risk as evidenced by the elevated screenings over a five-year period. This seems to be more of an issue for Nevis patients than St. Kitts patients. These results would suggest that resources should be dedicated to NCD self-management, frequent monitoring and group support [20].

Metabolic screening tools such as HBA1C and cholesterol, fasting blood glucose levels may be cost prohibitive to offer on a large scale. However, strategic and consistent evaluation of low costs risk screening such as regular calculations of BMI, and monitoring of blood pressure as a risk factor for both hypertension and diabetes, and recording family medical history could serve as the first risk assessment tools [20].

A most interesting finding is that though women outnumber men 3:1 in the study, their rates of diabetes were comparable to the men who were seen in the health centers. This calls into question the published propensity of women to be at higher risk of diabetes than men for diabetes in Caribbean studies. Perhaps the overrepresentation of women of African descent in general and Caribbean descent specifically, as diabetics is more tied to health seeking behavior associated with gender. As an example of a pending question, do the HC hours of operation (primarily 8:00 AM - 4:00 PM) influence male and female utilization patterns? We suggest further studies to investigate queries of this nature.

With an eye toward primary prevention, an assessment of the non-diabetic population would suggest “younger” age cohorts experience risk factors noted in the literature confirming a burgeoning population at risk of acquiring diabetes. It is worth noting that though the age cohort with the most cases of diabetes was 45–64, all of the age cohorts in this sample had individuals with diabetes diagnoses.

Currently, youth under the age of 15 constitute 27% of the nation’s population. As such, it is recommended that robust research be conducted to determine if the literature-based predictions of type 2 diabetes in youth are in fact true for St. Kitts and Nevis. Additionally, 97.8% of the population over 15 have completed 6 years or more of formal education and are functionally literate. Therefore, health education materials disseminated using traditional and innovative platforms across the Federation are integral in reducing the type 2 diabetes risk [16]. In light of the population density we suggest investment in health education curriculum and Ministry of Health interventions targeting youth to prevent widespread diabetes diagnoses, which is consistent with the Ministry’s focus on preventative population health interventions.

## Figures and Tables

**Table 1:** Metabolic Risk Ranges as defined by CARPHA.

Screening Tool	CARPHA Risk Ranges
Blood Pressure	≥ 130/85 mmHg
Fasting Blood Glucose	Prediabetes 100–125 mg/dL Diabetes ≥ 126 mg/dL
Resting Blood Sugar	> 200 mg/dL
HbA1c	≥ 6.5 mg/dL
Triglycerides	≥ 150 mg/dL
Combined Cholesterol	> 200 mg/dL

Adapted from: Caribbean health Research Council & Office of Caribbean Coordination of Pan American Health Organization [3].

**Table 2:** Demographics by Island.

Data Source	Age Range	Charts Reviewed	Gender	
			Male	Female
Nevis HCs (n = 439)	18–24	61	15	46
	25–34	78	24	54
	35–44	96	24	72
	45–64	147	43	104
	65–75	57	24	33
			**130**	**309**
St. Kitts HCs* (n = 322)	18–24	45	14	28
	25–34	59	16	41
	35–44	40	10	30
	45–64	138	44	94
	65–75	40	17	22
			**101**	**215**
Total (n = 761)	18–24	106	29	74
	25–34	137	40	95
	35–44	136	34	102
	45–64	285	87	198
	65–75	97	41	55
			231	524

* six St. Kitts charts gender of the patient was not specified; %n women Nevis n = 58.96% SKN n = 41.03% women federation total n = 68.85%; %n men Nevis n = 56.27% SKN n = 43.72% men federation total n = 30.35%.

**Table 3:** Diabetic patients with risk range screenings.

Data Source	Diabetic First Degree Relative	BP	FBG	CC	HBA1c
Nevis (n = 75)	31	67	48	32	30
St. Kitts (n = 98)	13	30	43	17	13
Total (n = 173)	44	97	91	49	43

% n Diabetic patients with risk screenings; Nevis n = 43.35%; St. Kitts n = 56.64%.

**Table 4:** Non-diabetics with elevated risk screenings.

Data Source	Diabetic First Degree Relative	BP	FBG	CC	HBA1c
Nevis (n = 364)	8	54	19	20	6
St. Kitts (n = 224)	96	64	7	18	2
Total (n = 588)	104	118	26	38	8

% n for non-diabetics with elevated risk screenings; Nevis n = 364 (61%); St. Kitts n = 224 (38%).
